# miR-622 is a novel potential biomarker of breast carcinoma and impairs motility of breast cancer cells through targeting NUAK1 kinase

**DOI:** 10.1038/s41416-020-0884-9

**Published:** 2020-05-18

**Authors:** Francesca Maria Orlandella, Raffaela Mariarosaria Mariniello, Peppino Mirabelli, Anna Elisa De Stefano, Paola Lucia Chiara Iervolino, Vito Alessandro Lasorsa, Mario Capasso, Rosa Giannatiempo, Maria Rongo, Mariarosaria Incoronato, Francesco Messina, Marco Salvatore, Andrea Soricelli, Giuliana Salvatore

**Affiliations:** 1 IRCCS SDN, Via Emanuele Gianturco 113, 80143 Naples, Italy; 2 Dipartimento di Scienze Motorie e del Benessere, Universita’ degli Studi di Napoli “Parthenope”, Via Medina 40, 80133 Naples, Italy; 3 CEINGE - Biotecnologie Avanzate S.c.a.r.l., Via Gaetano Salvatore 486, 80145 Naples, Italy; 4 Dipartimento di Scienze Biomediche Avanzate, Universita’ “Federico II”, Via Pansini 5, 80131 Napoli, Italy; 5 Dipartimento di Medicina Molecolare e Biotecnologie Mediche, Università degli Studi di Napoli “Federico II”, Naples, Italy; 6 Ospedale Evangelico Betania, Via Argine 604, 80147 Naples, Italy

**Keywords:** Cancer, Breast cancer

## Abstract

**Background:**

Aberrant expression of microRNAs (miR) has been proposed as non-invasive biomarkers for breast cancers. The aim of this study was to analyse the miR-622 level in the plasma and in tissues of breast cancer patients and to explore the role of miR-622 and its target, the NUAK1 kinase, in this context.

**Methods:**

miR-622 expression was analysed in plasma and in tissues samples of breast cancer patients by q-RT-PCR. Bioinformatics programs, luciferase assay, public dataset analysis and functional experiments were used to uncover the role of miR-622 and its target in breast cancer cells.

**Results:**

miR-622 is downregulated in plasma and in tissues of breast cancer patients respect to healthy controls and its downregulation is significantly associated with advanced grade and high Ki67 level. Modulation of miR-622 affects the motility phenotype of breast cancer cells. NUAK1 kinase is a functional target of miR-622, it is associated with poor clinical outcomes of breast cancer patients and is inversely correlated with miR-622 level.

**Conclusions:**

miR-622/NUAK1 axis is deregulated in breast cancer patients and affects the motility phenotype of breast cancer cells. Importantly, miR-622 and NUAK1 hold promises as biomarkers and as targets for breast cancers.

## Background

Breast cancer is the most commonly diagnosed cancer and the second leading cause of cancer death among women.^1^ Many factors are able to affect the development and progression of breast cancer, such as age, lifestyle, genetic factors, mammographic breast density, therapeutic radiation, age at menarche and at menopause, proliferative breast lesions, diabetes and obesity.^2,3^

The two most common morphological types of breast cancer are ductal and lobular carcinoma, while medullary breast carcinoma is a rare subtype of invasive breast carcinoma.^4^ Moreover, breast cancer is a highly heterogeneous disease further classified on the basis of different gene expression patterns, into different subtypes such as: basal-like, claudin-low, human epidermal growth factor receptor 2 (HER2)-enriched, luminal A, luminal B and normal-like.^5^ The triple-negative breast cancer (TNBC), characterised by negativity for oestrogen receptor (ER), progesterone receptor (PR) and HER2 expression, represents the most aggressive form and is associated with low survival, metastasis, recurrence, and development of chemo-resistance.^6,7^

Given the wide variety of molecular and pathologic diversity, breast cancer patients have different clinical outcomes and sensitivity to tumour therapies.^8^ For these reasons, further studies are still needed to explore novel molecular biomarkers and therapeutic targets for these patients. In particular, analysis of new prognostic non-invasive biomarkers is important to discriminate patients with different prognoses and to identify new anti-cancer treatment strategy. In recent decades, several studies unveil that microRNAs (miR) are stable and consequently detectable in the plasma of patients. Consequently, the analysis of circulating miRNAs could be important to uncover novel biomarkers for breast cancer.^9^

Increasing evidence suggests that miR-622 acts as a tumour suppressor in several types of human cancer such as glioma,^10,11^ gastric,^12,13^ pancreatic,^14^ hepatocellular^15^ thyroid^16^ and oesophageal squamous cell carcinomas,^17^ where it affects cell proliferation, migration and metastasis. Additionally, miR-622 suppresses migration and invasion of colorectal cancer cells by targeting K-RAS^18^ and DYRK2.^19^ In lung cancer, miR-622 is able to inhibit cancer metastasis by suppressing HIF-1α.^20^ In glioma miR-622 targets YAP^11^ and in breast cancer targets RNF8.^21^ Finally, a recent paper reported that in renal cell carcinoma, miR-622 suppressed cancer progression by targeting CCL18.^22^

Despite the accumulating evidence on miR-622 role in human tumorigenesis, its role in breast cancer remains, to our knowledge, not fully understood.

Here, we showed that miR-622 is downregulated in the plasma and in tissue samples of breast cancer patients where it acts as a tumour suppressor by reducing cell migration and invasion through targeting NUAK1 kinase. In conclusions, our findings provide evidence that miR-622 and NUAK1 are potential novel biomarkers and targets for breast cancer.

## Methods

### Clinical samples

Plasma samples were obtained from *n* = 17 age and race-matched healthy controls (HS) and *n* = 39 ductal invasive breast cancer patients, of which luminal A (*n* = 11), luminal B (*n* = 17), HER2-enriched (*n* = 2) and TNBC (*n* = 9). Moreover, from 20 patients we also collected breast cancer tissues and their adjacent normal tissues. For this study, we selected a group of naïve breast cancer patients that did not receive neoadjuvant therapy before blood sampling and surgery. All subjects were enrolled at the Ospedale Evangelico Betania (Naples, Italy). Samples processing started within 1 h from the collection, and all aliquots were stored at the SDN biobank (Naples, Italy) until use.^23^ This study was approved by the Ethics Committee of IRCCS Pascale (Naples, Italy) (Protocol n. 1/16 OSS SDN). Written informed consent was obtained from all subjects. This retrospective study was conducted anonymously and conforms to the principles of the Helsinki Declaration.

### Cell cultures

The breast cancer cell lines, MCF-7 and MDA-MB-231 were purchased from Leibniz-Institut DSMZ-Deutsche Sammlung von Mikroorganismen und Zellkulturen GmbH (Braunschweig Germany).

MCF-7 cell line was maintained in culture with Roswell Park Memorial Institute Medium (RPMI) (Thermo Fisher Scientific, Waltham, USA) supplemented with 10% of foetal bovine serum (FBS), L-glutamine, pyruvate sodium and human recombinant insulin (Thermo Fisher Scientific). MDA-MB-231 cells were maintained in culture in Dulbecco’s Modified Eagle Medium (DMEM) (Thermo Fisher Scientific) with 20 % of FBS and L-glutamine (Thermo Fisher Scientific).

### RNA extraction and q-RT-PCR

From plasma and from formalin-fixed paraffin-embedded (FFPE) tissue sections, total RNA was extracted using the miRNeasy Serum/Plasma and RNeasy FFPE Kits, respectively (Qiagen, Crawley, West Sussex, UK). During plasma extraction, Spike-in control (*C. elegans* miR-39 miRNA mimic, Qiagen) was added as an internal control according to the manufacturer’s protocol.

For reverse transcription of total RNA containing miRNA, cDNA was synthesised using a miScript II RT kit (catalogue number 218161) together with miScript HiSpec Buffer (for mature miRNA detection only) purchased from Qiagen.

Quantitative real-time PCR (q-RT-PCR) was performed using miScript Primer Assays (catalogue number 218300) specific for miR-622 expression (ID MS00005117) with the miScript SYBR Green PCR Kit (catalogue number 218073) (Qiagen).

The Ct-value of miR-622 was technically normalised with miR-16-5p (ID MS00031493, Qiagen)^24,25^ or with the snU6 (ID MS00029204, Qiagen) used as endogenous controls for plasma and FFPE tissue sections, respectively.

For the calculation of circulating level of miR-622, the fold changes of miR-622 were calculated with the formula: 2^−(sample 1 ΔCt − sample 2 ΔCt)^,^26^ where sample 1 represents each single plasma of breast cancer patient and sample 2 is the average of all (*n* = 17) healthy subjects controls.

From cell culture, total RNA was extracted using the mirVana^TM^ miRNA Isolation kit (Thermo Fisher Scientific) according to the manufacturer. RNA quantity was determined through NanoDrop spectrophotometer (Thermo Fisher Scientific). NUAK1 (Hs00934234_m1) and the endogenous control Human ACTB (β-ACTIN) (Hs01060665_g1) were evaluated with TaqMan assay kit (Thermo Fisher Scientific). The Ct values of each gene were performed in triplicate and the gene expression levels were calculated using the formula 2^−(sample 1 ΔCt − control ΔCt)^ ^26^ where the control ΔCt is represented by the cell lines transfected with the control plasmid (miR-Null or Anti-miR-Null) placed equal to one.

### Transfection

Breast cancer cell lines were stably transfected with a plasmid expressing pre-miR-622 (pEP-hsa-miR-622) and the corresponding empty vector (pEP-miR, named miR-Null) or with the hsa-miR-622 inhibitor (named Anti-miR-622) and the control vector (named Anti-miR-Null) purchased from GeneCopoeia (Nivelles, Belgium). Forty-eight hours after transfection, cells were selected in puromycin (Sigma-Aldrich, St. Louis, MO, USA) and miR-622 expression was evaluated by q-RT-PCR. One mass population for each cell line was selected on the basis of the miR-622 level and used for all experiments.

For transient transfection, NUAK1 plasmid, expressing NUAK1 mRNA without the 3′UTR, the relative control (Empty vector) and YAP plasmid were purchased from GeneCopoeia.

All transfections were performed using Lipofectamine 2000 (Thermo Fisher Scientific) according to the manufacturer’s instructions.

### Luciferase assay

MDA-MB-231/miR-622 and MDA-MB-231/miR-Null cells were plated in 96-well plates and transfected using FuGENE reagent with the pLightSwitch-NUAK1-3′UTR plasmid (catalogue number S814085) in which the 3′ untranslated region (UTR) of NUAK1, containing the putative binding site for miR-622, was cloned downstream of luciferase reporter gene. After 24 h, luciferase activity was measured according to the manufacturer’s protocol. All the reagents and appropriate control plasmids were purchased from Switchgear Genomics (La Hulpe, Belgium).

Deletion of the 3′-untranslated region (UTR) of NUAK1 was introduced into wild type plasmid using the QuikChange site-directed mutagenesis kit (Agilent Technologies, Santa Clara, CA) and the following oligonucleotides:

NUAK1-3′UTR-del Forward: 5′-ctctttgctggctgtgacagactgaaaaaggattgg-3′;

NUAK1-3′UTR-del Reverse: 5’-ccaatcctttttcagtctcacagccagcaaagag-3′.

### Migration assays

Wound healing and Transwell assays were performed to evaluate the effect of miR-622 on cell migration ability as previously described.^27^

Briefly, for wound healing assay, 3 × 10^5^ cells were seeded in 6-well plates, a wound was inflicted on confluent cell monolayer and closure was monitored at different time point. The wound area was measured through Cell^A^ software (Olympus Biosystem GmbH) and expressed as relative wound closure respect to control cells.

For Transwell assay, 1 × 10^5^ cells were seeded into the upper chamber containing polycarbonate membrane filter (Costar, Cambridge, MA, USA). Into the lower well, 500 μl of DMEM 20 % FBS was added as a chemo-attractant. After 24 h, migrated cells were fixed and quantified at optical density (O.D.) with the Microplate Reader (Model 550, Ultramar Microplate Reader, Bio-Rad).

### Matrigel invasion assay

Invasion assay was performed according to standard protocols. Briefly, 1 × 10^5^ cells were plated on a reconstituted extracellular matrix (Matrigel, BD Biosciences, San Jose, CA) on the upper chamber of Transwell (Costar). After 24 h, invaded cells were coloured with crystal violet and quantified at O.D. 550 nm.

### Matrigel 3D assay

Briefly, 150 μl of Basement Membrane Matrix (BD Biosciences) was plated on chambered coverglass (Nunc Lab-Tek, Sigma-Aldrich) according to the manufacturer’s instruction and, after 30 min, 5 × 10^4^ cells were plated and photographed at different time point.

### Proliferation assay

The number of viable cells was determined through tetrazolium compound [3-(4,5-dimethyl-2-yl)-5-(3-carboxymethoxyphenyl)-2-(4-sulfophenyl)-2H-tetrazolium, inner salt (MTS) reagent (CellTiter 96® AQueous One Solution Assay, Promega, WI, USA). Briefly, 1 × 10^3^ cells were plated in triplicate into 96-well culture plates; 20 µl of MTS was added to each well at different time points and then the absorbance at O.D. 490 nm was recorded.

### Western blot

Protein studies were carried out according to standard procedures. Anti-NUAK1 (#4458) and anti-YAP (#14074) antibodies were purchased from Cell Signaling Technology (Beverly, USA) while anti-α-TUBULIN (T9026) monoclonal antibody was purchased from Sigma-Aldrich. Secondary anti-mouse and anti-rabbit antibodies coupled to horseradish peroxidase were obtained from Bio-Rad. Enhanced chemiluminescent visualisation was obtained with enhanced chemiluminescence detection kit (Thermo Fisher Scientific).

### Analysis of public data sets

To assess the relationships between NUAK1 and miR-622 expression levels we used public data sets deposited in Gene Expression Omnibus (GEO) database. Public data were also used to investigate relapse-free survival (RFS) and disease-free survival probabilities.

NUAK1-dependent RFS in breast cancer subtypes was performed with the web-tool “Kaplan Meier Plotter”^28^ a data repository and analysis portal, which allows meta-analysis based biomarker assessments by merging and normalising gene expression and clinical data of numerous data sets.

GSE21653 dataset^29^ containing 266 medullary breast cancers was used to correlate NUAK1 and miR-622 expression and to perform disease-free survival analysis based on NUAK1 gene expression.

We used the GSE1456 dataset^30^ containing 159 breast cancers, to evaluate the correlations between NUAK1 and miR-622 expression in different breast cancer subtypes (basal, *n* = 25; ERBB2, *n* = 15; luminal-A, *n* = 39; luminal-B, *n* = 23; no subtype, *n* = 20; normal-like, *n* = 37). With the same purpose, we implemented the 17 normal breast tissues in GSE42568.^31^

All the GEO data sets we queried were obtained on an Affymetrix U133P2 chip. The R2 web platform (http://r2.amc.nl) was used to retrieve and download gene expression and associated clinical data.

All the analyses were performed within the R environment for statistical computing R Core Team (2016). (R: A language and environment for statistical computing. R Foundation for Statistical Computing, Vienna, Austria. URL: https://www.R-project.org/). For survival analysis, we used the “survival” package, which implements a log-rank test to assess statistical significance that was set at 5 %. Patients were split in “Low” and “High” based on NUAK1 median gene expression level.

In addition, The Cancer Genome Atlas (TCGA) repositories were screened for the survival analysis in different breast cancer subtypes characterised by low or high miR-622 expression levels. To this aim, Kaplan Meier survival plots, hazard rates with 95 % confidence interval and log-rank p-values were calculated to validate the prognostic value of miR-622 using the http://kmplot.com/analysis.^28^

### Statistical analyses

Statistical analyses were carried out using GraphPad Prism 6 software (La Jolla, CA). The Mann–Whitney non-parametric test was performed to analyse miR-622 expression level between two different groups. Kruskal–Wallis test was used to assess the association between miR-622 expression level and multiple comparisons. P values were determined by Student’s Unpaired t-test (two-tailed) and considered to be statistically significant when *p* < 0.05.

For power calculation, the sample size of 48 samples is sufficient. It was calculated considering a comparison between the averages of the measurements in the patient groups with a Mann–Whitney test, with an “effect size” *d* = 0.75, a power of 80% and α equal to 0.05 (Supplementary Fig. 1).

## Results

### miR-622 is downregulated in plasma and in tissues of breast cancer patients

**Fig. 1:**
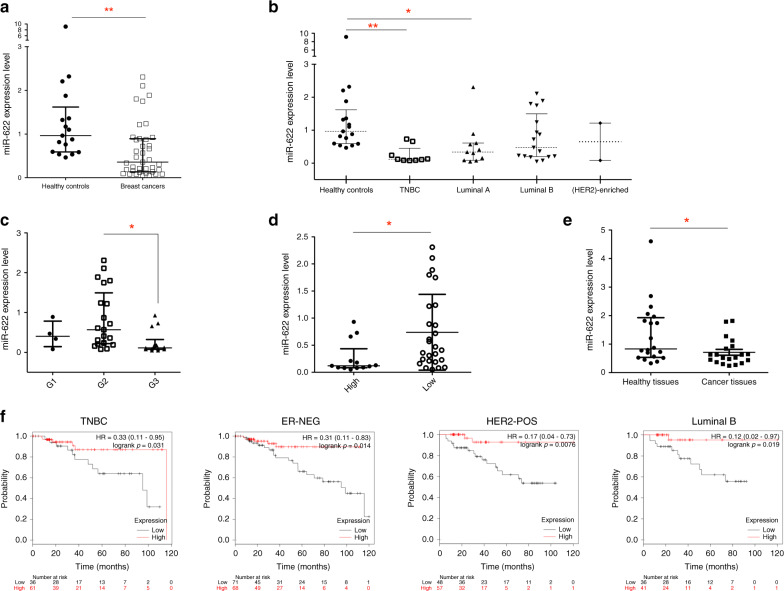
miR-622 is downregulated in plasma and in tissues of breast cancer patients. Relative miR-622 expression level was analysed by q-RT-PCR in the plasma of healthy controls (*n* = 17) compared to the plasma of all breast cancer patients (*n* = 39) (**a**) or of breast cancer specific subtypes (**b**). Correlations between plasma miR-622 expression levels and tumour grade (**c**), and Ki67 expression (low <30%; high ≥30%) (**d**) in breast cancer patients. **e** Relative expression level of miR-622 in breast tumour formalin-fixed paraffin-embedded tissues compared to respective adjacent non**-**tumour tissues (*n* = 20). Each experiment was performed twice in triplicate and data are expressed as median with interquartile range. **f** Kaplan–Meier survival plot of miR-622 in the triple-negative breast cancer (TNBC), in oestrogen receptor (ER)-negative, epidermal growth factor receptor 2 (HER2 pos) and in luminal B samples using the TCGA dataset. Hazard ratio (HR) values for miR-622 and overall survival (OS Probability) and log-rank *p* values are reported. *p < 0.05; **p < 0.01.

Since the differential expression of specific miRNAs in plasma of breast cancer patients could be used a diagnostic biomarker,^32,33^ we collected plasma of 39 females affected by ductal breast carcinoma and of 17 normal healthy subjects used as controls and performed q-RT-PCR to determine miR-622 level. As shown in Fig. 1a, miR-622 expression was significantly lower in the plasma of all breast cancer patients in comparison to the plasma obtained from healthy control subjects. In detail, by analysing specific breast cancer subtype in this dataset, we found that the plasma level of miR-622 was significantly decreased in the TNBC and in the luminal A subtypes (Fig. 1b). Furthermore, to investigate a possible role of miR-622 in the aggressive behaviour of breast cancer pathogenesis, we looked for the correlation between miR-622 plasma levels and clinicopathological features (summarised in Supplementary Table 1) of the patients analysed and we found that miR-622 expression inversely correlated with advanced tumour grade (G3) and high Ki67 level (30%) (Fig. 1c, d).

To determine if it’s downregulation occurs also in tissues, we measured miR-622 expression level in 20 FFPE breast cancer tissues and in their pair-matched adjacent normal tissues collected from the same patients in which we analysed miR-622 plasma level. As shown in Fig. 1e, miR-622 was significantly lower in FFPE tissues of breast cancer patients respect to adjacent normal tissues, suggesting that miR-622 downregulation could be related to breast cancer cells and not to the stromal cells of the tumour microenvironment.

In addition, TCGA data collection were used to assess the prognostic value of miR-622 in different breast cancer subtypes. As shown in Fig. 1f, Kaplan–Meier survival plot, reporting the hazard ratios (HRs) and *p*-values (log-rank test) showed that in the two patient cohorts (with high or low miR-622 level), high level of miR-622 is significantly correlated with overall survival in TNBC (*n* = 97; *p* = 0.031), in ER-negative (*n* = 139; *p* = 0.014), in HER2-positive (*n* = 105; *p* = 0.0076) and in luminal B (*n* = 77; *p* = 0.019) patients.

Collectively, our results suggest a role of miR-622 downregulation in the clinical outcome of breast cancer patients.

### miR-622 induces a reduction of NUAK1 expression by direct targeting its 3’UTR

**Fig. 2:**
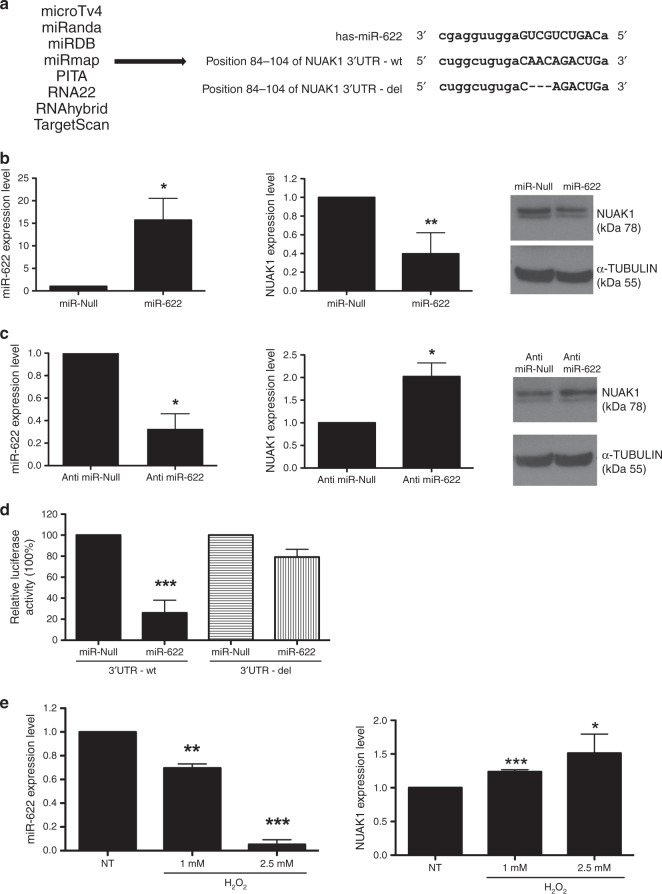
NUAK1 is a direct target of miR-622. **a** Shown is list of bioinformatics programs used to find the putative target of miR**-**622 (left) and the alignment of 3′UTR-NUAK1 wild type (−wt) and deleted (−del) version with the predicted binding site of miR-622 (right). **b**, **c** Expression levels of miR-622 (left) and NUAK1 (centre) mRNA were analysed by q-RT-PCR in MDA-MB-231 after transfection with miR-622 (**b**) or with Anti-miR-622 (**c**) respect to relative controls. β-ACTIN was used for normalisation. In **b**, **c** right, NUAK1 protein level was analysed in these cells by western blot, TUBULIN protein level was used as an endogenous control. **d** Luciferase activity was performed in MDA-MB-231/miR-622 and control cells co-transfected with NUAK1 3′UTR-wt or with 3′UTR-del. **e** MDA-MB-231 was treated with different doses of H_2_O_2_ and after 24 h, q-RT-PCR was performed to analyse miR-622 (left) and NUAK1 (right) expression levels. Each bar represents the mean ± SD of independent experiments. *p < 0.05; **p < 0.01; ***p < 0.001.

We interrogated several combined computational algorithms to predict the putative targets of miR-622. Using MicroTv4, MiRanda, miRDB, miRmap, PITA, RNA22, RNAhybrid and TargetScan, we unveil that the human 3′ untranslated region (UTR) of NUAK1 mRNA contains the sequences complementary to miR-622 (Fig. 2a). NUAK1 is a serine/threonine-protein kinase involved in various physiological and pathological processes^34^ and associated with the invasive and metastatic potential of human breast cancer cells.^35^

Based on the data accumulated, we explored whether miR-622 affected NUAK1 expression in the MDA-MB-231 breast cancer cell line. To achieve this goal, MDA-MB-231 cells were stably transfected with miR-622 or with miR-Null plasmids and the transfection efficiency was analysed by q-RT-PCR (Fig. 2b, left). As shown in Fig. 2b the expression of NUAK1 mRNA (2b, centre), and protein (2b, right) was decreased in MDA-MB-231/miR-622 respect to control cells (miR-Null).

In an opposite manner, when MDA-MB-231 were stably transfected with the inhibitor of miR-622 (named Anti-miR-622) (Fig. 2c, left), an increase of NUAK1 at mRNA and protein levels occurred (Fig. 2c, centre and right respectively).

To formally prove that miR-622 induces a reduction of NUAK1 expression by direct targeting its 3′UTR, we performed a luciferase activity assay. Thus, MDA-MB-231/miR-622 and MDA-MB-231/miR-Null cells were transiently transfected with a construct in which the 3’UTR of NUAK1 was inserted downstream of a luciferase reporter gene. We also transfect MDA-MB-231/miR-622 and MDA-MB-231/miR-Null cells with a plasmid in which the binding site for miR-622 in the 3’UTR was deleted (3’UTR-del).

The sequences of 3′UTR -wt and -del are reported in Fig. 2a. As shown in Fig. 2d, 24 h after transfection, relative luciferase activity was significantly lower in MDA-MB-231 co-transfected with miR-622 and with the 3’UTR -wt of NUAK1 respect to MDA-MB-231/miR-Null. Moreover, there was no significant difference in relative luciferase activity between MDA-MB-231/miR-622 and MDA-MB-231/miR-Null cells after transfection with a mutant version of this plasmid (NUAK1 3’UTR-del).

Taken together, our novel findings demonstrated that NUAK1 is a direct target of miR-622 in breast cancer cells.

### Insight in miR-622/NUAK1 axis

Since NUAK1 is a key component of the antioxidant stress response pathway,^36,37^ we investigated if the induction of oxidative stress modulates miR-622/NUAK1 axis in breast cancer. To this aim, we treated MDA-MB-231 cells with different doses of hydrogen peroxide (H_2_O_2_) and q-RT-PCR was performed to analyse the miR-622 expression level. As reported in Fig. 2e (left), H_2_O_2_ treatment significantly reduced the expression of miR-622 in a dose-dependent manner in comparison to untreated cells. Accordingly, the identified target of miR-622, NUAK1, is slightly increased in treated cells (Fig. 2e right).

Additionally, we examined a possible connection between YAP, miR-622 and NUAK1 in breast cancer cells, given recent studies linking NUAK family to Hippo pathway.^38^ To this aim, MDA-MB-231 cell line was transiently transfected with YAP plasmid or with empty vector and the expression of miR-622 and NUAK1 was analysed after 48 h. As shown in Supplementary Fig. 2, forced expression of YAP induced a reduction of miR-622 and an increase of NUAK1 levels.

Finally, Supplementary Fig. 3 shows the direct and indirect interactors of NUAK1 identified with three different public available programs: SIGNOR (https://signor.uniroma2.it),^39^ STRING (https://string-db.org)^40,41^ and Genemania (https://genemania.org).^42^

### NUAK1 is inversely correlated with miR-622 expression and with clinical outcomes of breast cancer patients

To uncover the association between miR-622 and NUAK1 expression in breast cancer patients, we consulted several public data sets, available from the Gene Expression Omnibus (GEO) database.

**Fig. 3:**
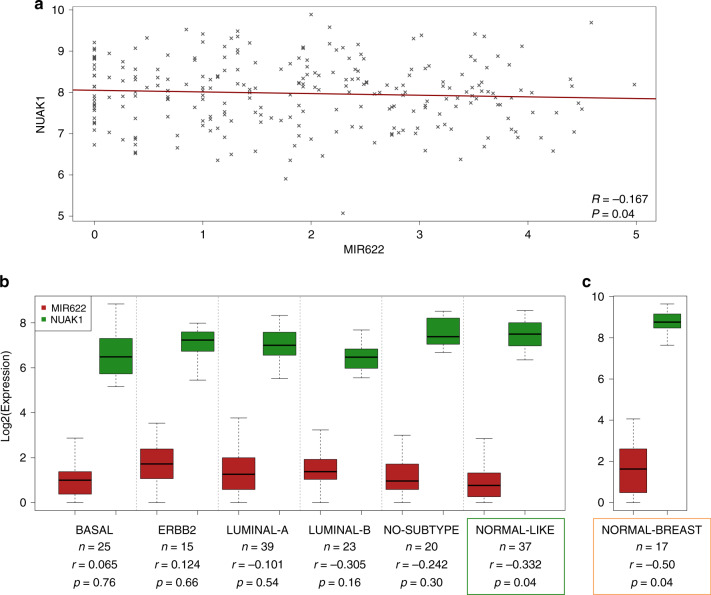
NUAK1 and miR-622 expression levels in breast cancer subtypes. **a** Scatter plot showing the inversely correlated expression of miR-622 (*X*-axis) and NUAK1 (*Y*-axis) in the GSE1456 dataset. The linear fit is shown in dark red. **b** The box plot show miR-622 and NUAK1 expression by tumour subtype in GSE1456 (basal, *n* = 25; ERBB2, *n* = 15; luminal-A, *n* = 39; luminal-B, *n* = 23; no subtype, *n* = 20; normal-like, *n* = 37). **c**. miR-622 and NUAK1 expression in 17 normal breast samples of GSE42568. Gene expression data were Log2 transformed before plotting. Correlation levels (*r*) were calculated with Pearson test. Statistical significance was assessed with *T*-test.

First, we consulted the GSE1456 dataset that includes the gene expression profiles of 159 population-derived breast cancer patients.^30^ As shown in Fig. 3a, correlation analysis unveils in this dataset a significant inverse correlation (*r* = −0.167; *p* = 0.04) between miR-622 and NUAK1 in breast cancer patients. Moreover, analysing miR-622 and NUAK1 expression in each breast tumour subtype of this dataset, we found that this inverse correlation occurs specifically in the normal-like subtype (*r* = −0.332; *p* = 0.04) (Fig. 3b).

Next, to improve our understanding of miR-622/NUAK1 axis, we consulted another dataset repository (GSE42568)^31^ and we found a significant inverse correlation (*r* = −0.50; *p* = 0.04) between miR-622 and NUAK1 also in 17 normal breast tissues (Fig. 3c) suggesting that miR-622 plays an important role in the maintenance of physiological NUAK1 expression in normal breast tissues.

**Fig. 4:**
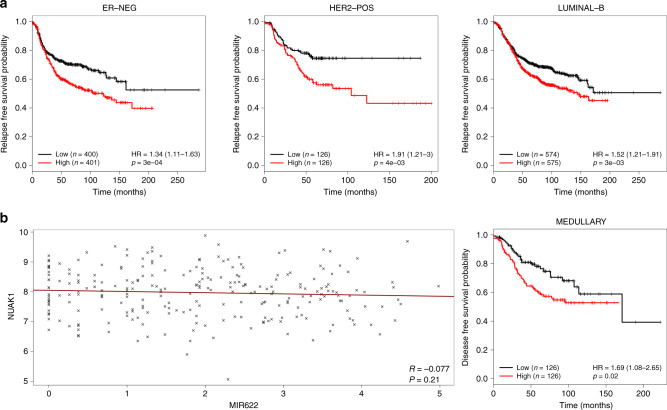
Relationships between NUAK1 expression and survival probabilities in intrinsic subtypes of breast cancer. **a** Kaplan–Meier curves showing the relapse-free survival probability in ER-negative, HER2-positive and luminal-B breast cancers subtypes for NUAK1. Patients were split in “Low” and “High” based on NUAK1 median gene expression level. Significance was assessed by Chi-Square test. **b** Medullary breast cancer (GSE21653). Left panel: scatter plot showing the inversely correlated expression of miR-622 (*X*-axis) and NUAK1 (*Y*-axis) (Pearson *r* = −0.077; *p* = 0.21). The linear fit is shown in dark red. Right: disease-free survival probability based on NUAK1 expression levels. Patients were split in “Low” and “High” based on NUAK1 median expression level. Expression data were Log2 transformed before plotting. Significance was assessed by log-rank test. Hazard Ratios and the respective 95% confidence interval are shown in parentheses.

Finally, survival meta-analysis was performed to estimate the overall- and relapse-free survival probabilities respect to NUAK1 expression. We found that low NUAK1 expression levels significantly correlates with better prognosis and relapse-free survival in some tumour subtypes: ER-negative (*p* = 0.00033), HER2-positive (*p* = 0.004) and luminal B (*p* = 0.003) subtypes (Fig. 4a). Thus, our data provide evidence to support the role of NUAK1 in predicting survival rate in breast cancer patients.

Analysing the GSE21653 database,^29^ we investigated NUAK1 and miR-622 expression also in medullary breast cancer patients, and Kaplan–Meier disease-free survival curve showed that low level of NUAK1 is significantly correlated with disease-free survival (*p* = 0.02) (Fig. 4b, right). However, in this dataset the inverse correlation between miR-622 and NUAK1 (*r* = -0.077), was not statistically significant (*p* = 0.21) (Fig. 4b, left).

### miR-622 modulates the migration ability of breast cancer cells

**Fig. 5:**
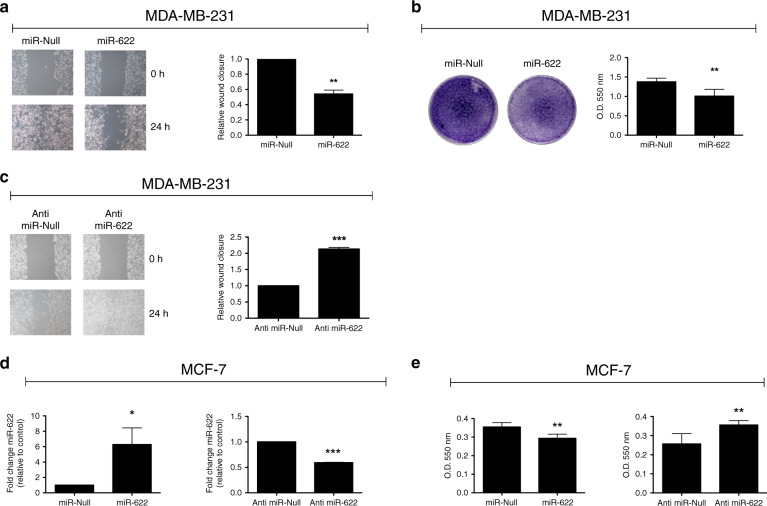
miR-622 affects migration of breast cancer cell lines. Cell migration ability was determined in MDA-MB-231/miR-622 cells by wound healing (**a**) and by Transwell migration (**b**) assays**. c** Wound healing was also performed in MDA-MB-231 transfected with Anti-miR-622 respect to control cells (Anti-miR-Null). **d** Expression level of miR-622 was monitored, by q-RT-PCR, in MCF-7 cell line after stable transfection with miR-622 (left) or with Anti-miR-622 (right) plasmids respect to relative control cells (MCF-7/miR-Null or MCF7/Anti-miR-Null). **e** Cell migration ability was determined by Transwell migration assay also in MCF-7 after stable transfection. Data are presented as the mean ± S.D. *p < 0.05; **p < 0.01; ***p < 0.001.

Next, we sought to investigate whether miR-622 has a tumour suppressor role in breast cancer cell lines. To this aim, we analyse the biological effects of miR-622 on cell migration ability performing wound healing and Transwell migration assays. As shown in Fig. 5a, b, forced expression of miR-622 in MDA-MB-231 cells induced a reduction of migration ability into the wound and into the Transwell inserts respect to control cells. In the opposite manner, when we stably silenced the expression of miR-622 in MDA-MB-231 cells, an increase of cell migration capability occurred (Fig. 5c).

To further confirm the effects of miR-622 on breast cancer cells migration, we also stably transfected the miR-622 precursor or its inhibitor (Anti-miR-622) into human breast adenocarcinoma cell line, MCF-7 (Fig. 5d, respectively left and right). In the generated cell lines, we examined the motility phenotype. As shown in Fig. 5e (left), miR-622 significantly reduced migration ability into the Transwell respect to control cells (MCF-7/ miR-Null). Conversely, stable silencing of miR-622 enhanced the migration ability respect to MCF-7/ Anti-miR-Null cells (Fig. 5e, right).

The stable modulation of miR-622 expression did not significantly affect the proliferation rate determined by MTS assays (Supplementary Figure 4).

Collectively, our data demonstrate that miR-622 is able to suppress the ability of breast cancer cells to migrate.

### miR-622 modulates the invasion ability of breast cancer cells

**Fig. 6:**
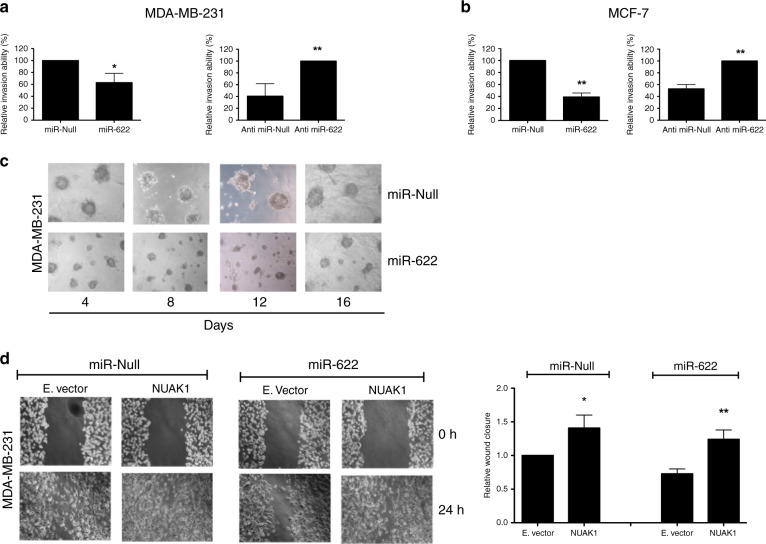
miR-622 affects cell motility in breast cancer cell lines through targeting NUAK1. **a** Matrigel invasion assay was performed in MDA-MB-231 cells transfected with miR-622 (left) or with Anti-miR-622 (right) and relative controls. After 24 h, eluted invading cells were quantified at 550 nm O.D. and reported as relative invasion ability (%). **b** Matrigel invasion assays were used to evaluate the invasion ability of MCF-7 after stable transfection with miR-622 (left) or Anti-miR-622 (right) and their relative controls. **c** Representative images of MDA-MB-231 transfected cells plated in Matrigel 3D at different time point. **d** After transient transfection of NUAK1 or Empty vector in MDA-MB-231/miR-Null and in MDA-MB-231/miR-622 cells, wound gaps were inflicted, photographed (left) and measured (right) at 0- and 24-h time points. Data shown are from two independent experiments ± S.D. *p < 0.05; **p < 0.01.

Next, to underscore the contribution of miR-622 on breast cancer invasion ability, Matrigel-coated membranes were used for invasion assays. As shown in Fig. 6a (left), the invasion ability of MDA-MB-231 was significantly lower when these cells were stably transfected with miR-622 compared to miR-Null. By contrast, depleting endogenous expression of miR-622 by stable transfection with Anti-miR-622 plasmid, increased the invasion ability of MDA-MB-231 cells (Fig. 6a, right). Coherently, also the modulation of miR-622 expression in MCF-7 cell line affected the invasion ability after stable transfection (Fig. 6b).

Finally, we also observed that after 4 days seeded in Matrigel 3D, MDA-MB-231/miR-622 cells appeared smaller respect to MDA-MB-231/miR-Null cells (Fig. 6c). However, apparently at 16 days, the spheroids appear to recover, suggesting that miR-622 slow-down and not halted the grow in Matrigel 3D.

Overall, these results demonstrate that miR-622 impairs tumour cell invasion and the aggressive behaviour of breast cancer cell lines.

### NUAK1 rescues the miR-622 induced phenotype in MDA-MB-231 cells

Having demonstrated that miR-622 is able to inhibit the motility phenotype of breast cancer cells, we next sought to determine whether the restoration of NUAK1 expression is able to revert the functions of miR-622. To this aim, we performed a wound closure assay in MDA-MB-231/miR-622 and in MDA-MB-231/miR-Null cells transiently transfected with a plasmid encoding NUAK1 mRNA deprived of its 3′UTR or with an Empty vector, used as a control. Figure 6d showed that NUAK1 increased the migration ability of MDA-MB-231/miR-Null cells. Importantly, NUAK1 deprived of its 3′UTR is able to revert the motile phenotype induced by miR-622, demonstrating that NUAK1 is also a functional target of miR-622.

## Discussion

Breast cancer represents the most common cancer among women and despite the significant advances in the early diagnosis, the development of more accurate prognostic biomarkers and of novel therapeutic strategies, still represents the main goal in cancer research.^43^

MicroRNAs (miRNAs) are non-coding molecules that negatively regulate gene expression by binding the complementary sequences of the 3′UTR of specific mRNA target.^44^

The aberrant expression of miRNAs is correlated with tumour progression and drug resistance in several human diseases including breast cancers.^45,46^ Moreover, several studies reported that intrinsic breast cancers subtypes are characterised by different molecular miRNA signatures.^47,48^

All these data and the evidence that miRNAs can be extracted and quantified in the plasma from cancer patients without degradation, suggest that miRNAs have a potential role as ideal biomarkers for diagnosis and prognosis of breast cancer patients.^9,49^

Furthermore, undoubtedly, miRNA-based therapies hold great promise, delivering antagomiRs or miRNA mimics specifically into the target cells by nanocarriers.^50–52^

Recently, it has been also indicated that specific set of miRNAs have important roles in endocrine resistance and in hormonal therapies contributing to the clinical benefits of breast cancer patients.^53^

The two most common types of breast cancer are ductal and lobular carcinoma. Since about 80% of all breast cancers are ductal carcinomas, we collected plasma from healthy controls and from ductal breast cancer patients at the time of diagnosis and we have shown that the expression of miR-622 is decreased in the plasma of these patients. Moreover, we also reported that miR-622 expression was inversely correlated with aggressive clinicopathological features as advanced tumour grade and high levels of Ki67, identifying the low expression level of miR-622 as a novel prognostic factor in patients with aggressive breast cancer.

Recently, many deregulated miRNAs have been identified in the stroma cells of tumour microenvironment where they regulate multiple signalling pathways modulating cancer development and progression.^54^ Here, we provided evidence that the low level of miR-622 in the plasma of breast cancer patients is likely due to its downregulation in breast cancer cells and not to stromal cells of the tumour microenvironment.

Furthermore, we showed that miR-622 tumour suppressor activity in breast cancer cells is mediated by direct targeting the NUAK1 kinase. The AMPK-related kinase family members, including NUAK1 (aka ARK5) and NUAK2 (aka SNARK),^55^ have been implicated in several physiological processes such as regulation of cell proliferation and gene transcription. Since NUAK1 and NUAK2 genes encodes for two proteins with the similar structural organisation in the catalytic domain,^56^ we verified if NUAK2 is also a predicted target of miR-622. However, by interrogating the several algorithms within the miRWalk program (http://zmf.umm.uni-heidelberg.de/apps/zmf/mirwalk2/), we found that the 3′UTR of NUAK2 is not one of the high score putative predicted target of miR-622.

NUAK1 is a serine/threonine kinase involved in cell adhesion, polarity and in epithelial-mesenchymal transition.^57^ NUAK1 overexpression is correlated with poor clinical outcome in various types of cancers.^34,58,59^

In this scenario, previous studies provided evidence that NUAK1 is closely involved in tumour progression of colon cancer,^60^ glioma,^61^ gastric,^62^ ovarian^63^ and nasopharyngeal carcinoma.^64^ Consistent with our study on breast cancer, Chang reported, through in vitro and in vivo experiments, that NUAK1 enhanced the invasive and metastatic potential of MDA-MB-231 cell line mediating AKT signalling.^35^

In the C-terminal catalytic domain of NUAK1 there is a site for liver kinase B1 (LKB1) phosphorylation and activation.^65^ After the activation, NUAK1 is able to control cell motility through the assembly and disassembly of cytoskeletal proteins.^66^ Importantly, LKB1 is a tumour suppressor kinase and its loss promotes breast cancer metastasis and invasion.^67,68^

Another important mechanism of regulation of NUAK1, described in cancer, is the oxidative stress pathway.^36,37^ Coherently, also in this study we have obtained evidence that this mechanism could be involved in the regulation of miR-622 and NUAK1 in breast cancer cells.

Additionally, we have examined a possible connection between YAP expression and miR-622 in breast cancer cells and found that YAP overexpression induced a reduction of miR-622 and an increase of NUAK1 levels. Interestingly, in a recent paper, Xu demonstrated that YAP is a direct target of miR-622 in glioma cells.^11^

Breast cancer is classified into several intrinsic subtypes on the basis of histological and molecular characteristics and distinct clinical outcomes.^7,69^ It is very interesting that we found an inverse correlation between miR-622 and NUAK1 in the normal-like subtype, characterised by the same status of the normal breast profiling but with poor clinical outcome.^5,69^ Consistent with our data, Riaz and colleagues analysed the difference in gene expression profiles between basal-like and normal-like/claudin-low breast cancer cell lines, and found the lower expression of miR-622 in normal-like subtype.^70^

Recently, Liu reported, from in silico analysis, that in all breast cancer patients of the TCGA dataset, survival rate was negatively correlated with high level of miR-622.^21^ However, we found that by analysing specific subtypes of breast cancers, high levels of miR-622 positively correlated with overall survival in the TNBC, ER-neg, HER2-pos and luminal B subtypes. These different results could be explained by the fact that breast cancer is an extremely heterogenous disease.

Given the importance of ER, PR and HER2 status as prognostic factors for breast cancer outcomes, we also studied the correlation between NUAK1 and RFS in each cancer subtype. Kaplan–Meier curves showed that NUAK1 represents a predictor factor of greater RFS, not in whole population of breast cancer patients, but only into specific subtypes: ER- negative, HER2-positive and luminal B subtypes. In addition, analysing the GSE21653 database, we also found that in medullary breast carcinoma low level of NUAK1 significantly correlated with a higher disease-free survival probability. Thus, our data provide evidence to support the role of NUAK1 as novel prognostic biomarker in predicting survival rate of specific subtypes of breast cancer patients.

Interestingly, the inverse correlation between miR-622 and NUAK1 in 17 normal breast tissues suggests that miR-622 key an important role in the maintenance of physiological NUAK1 expression level in normal tissues.

The 3’UTR of NUAK1 is a target of multiple miRNAs deregulated in different types of human cancers. In non-small cell lung carcinoma (NSCLC) and in hepatocellular cancer, NUAK1 is negatively regulated by miR-204,^71,72^ in pancreatic cancer NUAK1 is regulated by miR-96^73^; while miR-203 suppresses cell invasiveness through targeting NUAK1 both in head and neck cancer^74^ and in squamous cell carcinoma.^75^ Recently, NUAK1 expression results enhanced following the downregulation of miR-145a-5p and of miR-30b-5p respectively in nasopharyngeal and prostate cancer.^76,77^ In intrahepatic cholangiocarcinoma NUAK1 is directly targeted by two different miRNAs: miR-145^78^ and miR-424-5p.^79^

In the Supplementary Table 2 is reported a list of all published miRNAs that regulates NUAK1 in human cancer along with their binding sequences and position. In addition, by using miRanda alghorithm (www.microrna.org) it is worth to note that miR-622 binding site in the 3′UTR of NUAK1 does not overlap with others miRNAs.

It has been demonstrated that NUAK1 could represent an attractive target for treatment of MYC-driven cancers.^80–82^ Deregulated level of the transcription factor MYC is also reported in breast cancer^83^ thus it is possible that MYC could influence the miR-622/NUAK1 pathway in breast cancers, providing a rationale for target therapy.

Moreover, it is described that WZ4003 (a dual inhibitor of NUAK1 and NUAK2) and HTH-01-015 (a selective inhibitor of NUAK1) are able to inhibit the phosphorylation at serine of myosin phosphate-targeting subunit 1 (MYPT1) induced by NUAK1.^84^ Banerjee also reported that WZ4003 and HTH-01-015 are able to inhibit migration and proliferation of mouse embryonic fibroblasts. These data suggest that further researches using specific NUAK1 inhibitors should be carried out to better characterise the biological roles and therapeutic potentials of the NUAK1 kinase in breast cancer.

Additionally, a recent paper reported that the inhibition of NUAK1 enhances cisplatin cytotoxicity in NSCLC cells suggesting that also in breast cancer NUAK1 could represent a novel target against drug resistance.^85^

In conclusion, we proposed that the miR-622/NUAK1 axis controls tumour cell migration and invasion of breast cancer cell lines and, relevantly, our results support the notion that miR-622 and NUAK1 kinase could have clinical utility both as predictive biomarkers and as therapeutic targets in breast cancer patients.

## Supplementary information

Supplementary information

## Data Availability

Publicly available data sets were consulted in this study (Gene Expression Omnibus, GEO database; http://kmplot.com/analysis/).
