# Supplementary material for: miR-622 is a novel potential biomarker of breast carcinoma and impairs motility of breast cancer cells through targeting NUAK1 kinase
Source: Br J Cancer. 2020 May 18;123(3):426–37. doi: 10.1038/s41416-020-0884-9 (PMC7403386; doi:10.1038/s41416-020-0884-9)
Supplement: Supplementary file 1 — Supplementary information [file 41416_2020_884_MOESM1_ESM.docx]

**Supplementary information**

***miR-622 is a novel potential biomarker of breast carcinoma and impairs motility of breast cancer cells through targeting NUAK1 kinase***

Francesca Maria Orlandella^1^, Raffaela Mariarosaria Mariniello^2,3^, Peppino Mirabelli^1^, Anna Elisa De Stefano^2,3^, Paola Lucia Chiara Iervolino^3,4^, Vito Alessandro Lasorsa^3,5^, Mario Capasso^1,3,5^, Rosa Giannatiempo^6^, Maria Rongo^1^, Mariarosaria Incoronato^1^, Francesco Messina^6^, Marco Salvatore^1^, Andrea Soricelli^1,2^, Giuliana Salvatore^1,2,3^

^1^ IRCCS SDN, Via Emanuele Gianturco 113, 80143 Naples, Italy.

^2^ Dipartimento di Scienze Motorie e del Benessere, Universita’ degli Studi di Napoli “Parthenope”, Via Medina 40, 80133 Naples, Italy.

^3^ CEINGE - Biotecnologie Avanzate S.c.a.r.l., Via Gaetano Salvatore 486, 80145 Naples, Italy.

^4^ Dipartimento di Scienze Biomediche Avanzate, Universita’ “Federico II”, Via Pansini 5, 80131 Napoli, Italy.

^5^ Dipartimento di Medicina Molecolare e Biotecnologie Mediche, Università degli Studi di Napoli "Federico II", Naples, Italy.

^6^ Ospedale Evangelico Betania, Via Argine 604, 80147 Naples, Italy.

**Corresponding author**: Giuliana Salvatore, Dipartimento di Scienze Motorie e del Benessere, Universita’ degli Studi di Napoli “Parthenope”, Naples, Via Medina 40, 80133 Naples, Italy; [giuliana.salvatore@uniparthenope.it](mailto:Giuliana.salvatore@uniparthenope.it); Ph: + 39-081-3737865, Fax: + 39-081-3737808.

**Supplementary Table 1:** Clinical-pathological features of breast cancer patients analyzed.

| Characteristics | Number of Cases  (Total n = 39) | miR-622 expression level (Median) | p value |
| --- | --- | --- | --- |
| Age at diagnosis  < 50  ≥ 50 | 12  27 | 0.20  0.41 | ns |
| Elston-Ellis grade  G1  G2  G3 | 4  21  14 | 0.40  0.83  0.11 | ***p* < 0.01** |
| Estrogen Receptor  +  - | 28  11 | 0.38  0.12 | ns |
| Progesterone Receptor  +  - | 28  11 | 0.38  0.12 | ns |
| HER2+ Receptor  > 10%  absent | 21  18 | 0.23  0.41 | ns |
| Ki67  low  high | 26  13 | 0.74  0.27 | ***p* < 0.015** |

ns: indicates not statistically significant results.

Ki67 is considered low <30%, high ≥ 30%.

For estrogen receptor and progesterone receptor the cut-off is 1% (based on St. Gallen guidelines Goldhirsh, 2013).

**Supplementary Table 2:** A summary of miRNAs directly targeting the 3’UTR of NUAK1 kinase in human cancer.

| miRNA | Cancer | | Binding sequences | Position | | References |  |
| --- | --- | --- | --- | --- | --- | --- | --- |
| miR-424-5p | | intrahepatic cholangiocarcinoma | UGCUGCU | 2913-2919 | Wu, Int J Biol Sci. 2019 | | |
| miR‐145‐5p | | nasopharyngeal  intrahepatic cholangiocarcinoma | AACUGGA | 1018-1024 | Lan, Cell Mol Med. 2019  Xiong, Bioche. Bioph Res Com. 2015 | | |
| miR-204 | | hepatocellular  non-small-cell lung  carcinoma | AAAGGGAA | 1958-1965 | Yu, Biochem Cell Biol. 2019  Shi, Br J Cancer. 2014 | | |
| miR-625 | | nasopharyngeal carcinoma | UUCCCCC | 1256-1263 | Chen, Onco Targets Ther. 2019 | | |
| miR-125b | | bladder cancer | UUCAGGG | 2943-2949 | Wang, J Cell Biochem. 2019 | | |
| miR-30b-5p | | prostate cancer | GUUUAC | 2769-2774 | Guan, Int J Clin Exp Pathol. 2018 | | |
| miR-203 | | head and neck cancer  squamous carcinoma | AUUUCA | 3218-3223 | Obayashi, Oncotarget. 2016  Benaich, Cell Rep. 2014 | | |
| miR-96 | | pancreatic cancer | UGCCAA | 3021-3026 | Huang, Int J Mol Med. 2014 | | |
| miR-211 | | melanoma | AAAGGGA | 1955-1961 | Bell, J Invest Dermatol. 2014 | | |

**Supplementary Figures**

**
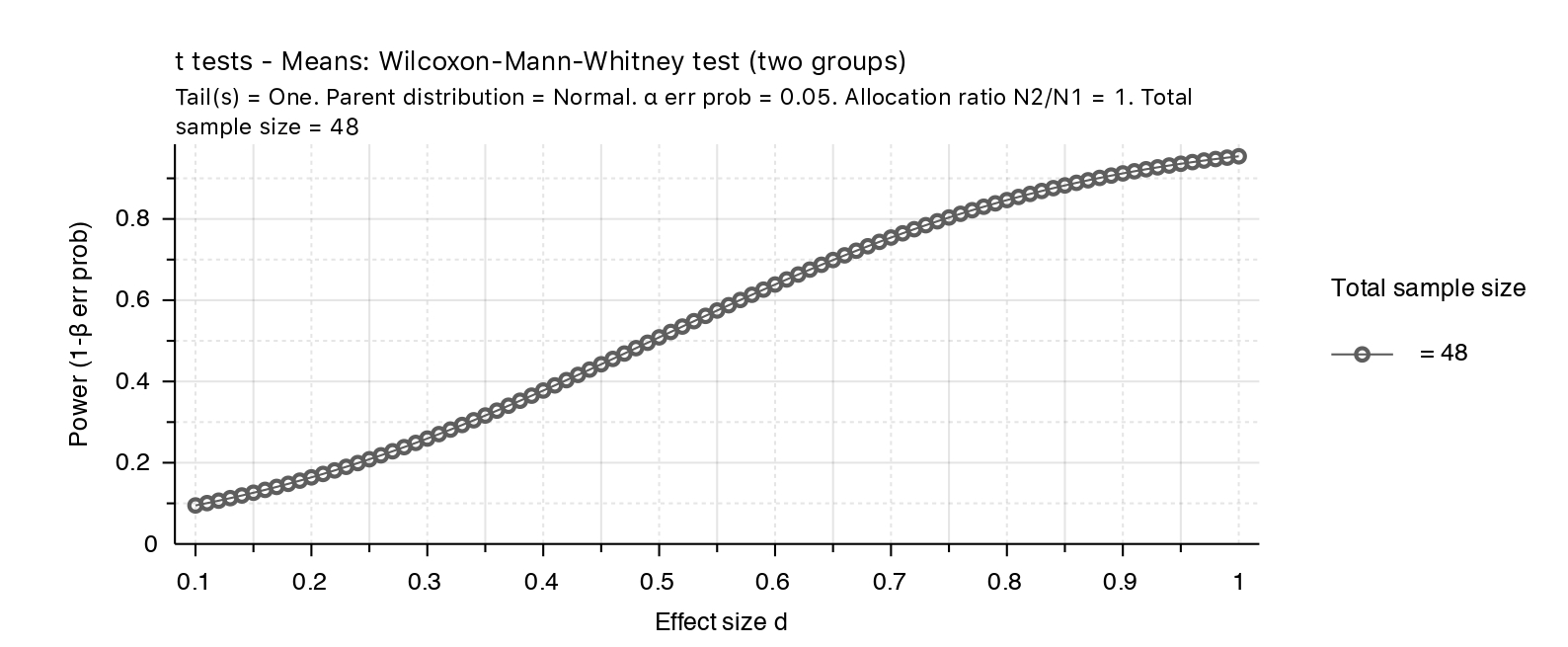
**

**Supplementary Figure 1.** **Power calculation analysis**. A power calculation was performed to determine the adequacy of sample size and calculated considering a comparison between the averages of the measurements in the patient groups with a Mann-Whitney test, with an "effect size" d = 0.75, a power of 80% and α equal to 0.05.

**

**

**Supplementary Figure 2. Insight on YAP miR622/NUAK1 axis. a.** MDA-MB-231 cells were transiently transfected with YAP plasmid or with control empty vector (E. Vector) and after 48 hours post transfection, YAP protein level was detected by western blot. α-TUBULIN was used as an endogenous control. **b-c.** Expression level of miR-622 (**b**) and of NUAK1 (**c**) in MDA-MB-231 cells was detected by q-RT-PCR in MDA-MB-231 transfected with YAP plasmid or with E. Vector. Columns indicate means ± S.D. * p < 0.05.

**
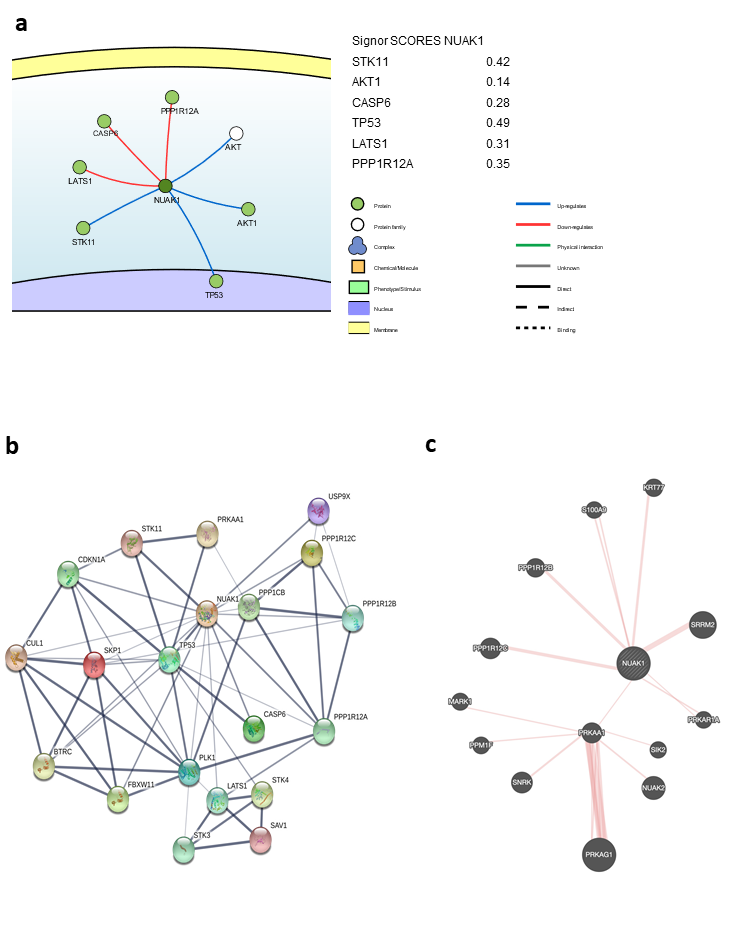
**

**Supplementary Figure 3. Identification of interactors of NUAK1 with open access web-tools.** To predict the protein-protein interactors of NUAK1 the following programs were used: (**a**) SIGnaling Network Open Resource (Signor). The table reports the computed scores between NUAK1 and its interactors. (**b**) STRING, reporting interactions (from text mining, experiments and databases) with confidence above 0.4. (**c**) GeneMania reporting only physical interactons between proteins.

**

**

**Supplementary Figure 4.** **Cell proliferation rate in breast cancer transfected cell lines**. **a.** Cell proliferation rate was monitored by MTS assay in MDA-MB-231 cell line transfected with miR-622 or with Anti miR-622 plasmids or with relative controls. **b.** Through MTS assay proliferation rate was also monitored in MCF-7 cell line after stable transfection with miR-622 or with Anti miR-622 plasmid in comparison to relative control cells. Data are presented as the mean ± S.D.
